# Synchronous mucinous adenocarcinoma of the rectosigmoid seeding onto a pre-existing anal fistula

**DOI:** 10.1186/1477-7800-3-25

**Published:** 2006-09-08

**Authors:** Nemandra Sandiford, Patsy R Prussia, Antonio Chiappa, Andrew P Zbar

**Affiliations:** 1 Professorial Department of Surgery, School of Medicine and Clinical Research, Queen Elizabeth Hospital, Barbados; 2 Department of Pathology Queen Elizabeth Hospital, Barbados; 3 Department of Surgery, European Institute of Oncology and University of Milano –, Italy

## Abstract

Carcinoma within a long-standing fistula-in-ano is rare and may be defined by specific neoplastic involvement of the fistulous track in the absence of rectal mucosal carcinoma. The presence of a carcinoma of mucinous histology occurring synchronously in the perianal region and the colon is exceptionally rare. We present a case with a review of the literature concerning its aetiopathogenesis and treatment. A 72-year-old man with a 2 months history of dark red rectal bleeding and mucus per rectum with alternating constipation and diarrhoea, was observed. Clinical examination and a barium enema showed a perianal fistula and an annular stenosing lesion of the rectosigmoid. Preoperative CT scan confirmed the colonic lesion. Colonic resection and wide fistula excision were performed. Histology showed an adenocarcinoma with a clear resection margins. The fistula also showed a similar histology. Chemoradiation (5-Fluorouracil (425 mg/m^2^) and Leucovorin (20 mg/m^2^) with 4500 cGy external beam radiotherapy was utilized. Subsequent clinical follow-up and CT examination of the patient has not revealed recurrent disease at 14 months.

## Background

Carcinoma within a long-standing fistula-in-ano is rare[1] and may be defined by specific neoplastic involvement of the fistulous track in the absence of rectal mucosal carcinoma. Such tumours are most frequently well-differentiated and mucinous in type, presenting late because of their insidious slow growing nature[2,3]. Due to their association with a symptomatic perianal fistula, malignant transformation is often initially overlooked[4]. The presence of a carcinoma of mucinous histology occurring synchronously in the perianal region and the colon is exceptionally rare[5,6]. We present such a case along with a review of the available literature concerning its aetiopathogenesis and treatment.

## Case report

A 72-year-old Afro-Caribbean male presented to our University Hospital with a 2 months history of dark red rectal bleeding and mucus per rectum with alternating constipation and diarrhoea as well as 20 lb. weight loss. There was no family history of colorectal neoplasia. There was an attendant history of perianal discharge for the previous 2 years and the patient volunteered that he had been seen in the United Kingdom and told that he had a perianal fistula in 1999. Clinical examination was unremarkable apart from the perianal area where a perianal fistula was evident which appeared to be low trans-sphincteric in nature. It was firm and indurated. A barium enema which had been performed outside the hospital showed an annular stenosing lesion of the rectosigmoid. Preoperative CT scan confirmed the colonic lesion but was otherwise unremarkable. In view of the presentation he was taken to the operating theatre after full bowel preparation for elective sigmoid colectomy and wide fistula excision.

**Figure 1:**
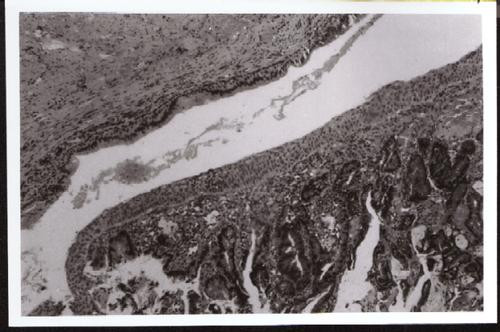
Section of the anal fistula track showing normal anal ductal epithelium (*above*) and infiltrating carcinoma (*below*). (× 100).

**Figure 2:**
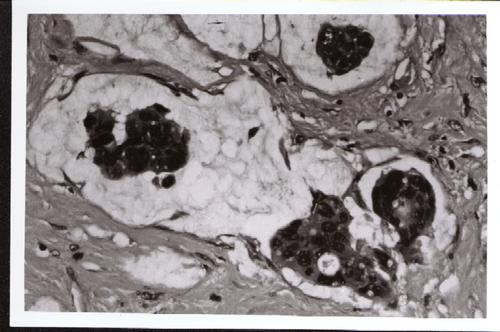
High-power view showing detached carcinoma cells floating within lakes of mucin. (× 400).

Histology of the colon showed a moderate to well differentiated adenocarcinoma with extensive mucinous components and none out of 10 lymph nodes resected was involved with tumour. (Stage IIA American Joint Committee Cancer) The resection margins of the tumour were clear. The fistula also showed a similar histology (Figure 1) with clear resection margins, involving the lower internal and external anal sphincter muscles associated with free floating dissociated tumour cells lying within lakes of mucin evident some distance from the epithelial lining. (Figure 2) The postoperative course of the patient was unremarkable and he has since undergone sandwich chemoradiotherapy utilizing 3 cycles of 5-Fluorouracil (425 mg/m^2^) and leucovorin (20 mg/m^2^) before and after 4500 cGy external beam radiotherapy (EBRT) to the perineum and inguinal portals. Clinical and CT examination of the patient has not revealed any signs of recurrent disease at 14 months.

## Discussion

Synchronous adenocarcinoma of the rectosigmoid and the perianum is exceptionally rare and we could only find 6 previously reported cases [5-10]. Very frequently, the symptoms in such patients are only attributed to their perianal disease and the development of supervening carcinoma is missed. It is therefore necessary to restress that it remains advisable in all cases of fistula and anorectal abscess to assess histology[11].

The presence of perianal malignancy of mucinous histology may have several aetiologies. One possibility is malignant degeneration within a long-standing anal fistula and this, although uncommon, has been well reported. The histology of such cases tends to be either squamous or mucinous, with Rosser first describing in 1931 the 3 basic criteria for definitional purposes which determine that a fistula has undergone malignant transformation[12]. For this diagnosis, the fistula should have been present for a minimum of 10 years, there should be no evidence of tumour within the rectal or anal canal mucosa and the internal opening of the fistula should be devoid of malignancy. A further clue to this diagnosis is the presence of free globules of mucin which lie away from areas of granulation tissue recognizable within a fistulectomy specimen, as noted in our case[13]. Of course, a rectal carcinoma may present as a fistula or with acute perirectal sepsis and it may be difficult in some cases to determine whether the tumour is a complication of a long-standing perianal fistula or whether the carcinoma itself has fistulated. Additionally, carcinoma may supervene within the rectum adjacent to a fistula in perianal Crohn's disease[14]. True cutaneous metastases to the anal skin, (from colonic or other types of carcinomas), are exceptionally rare; most likely resulting from retrograde vascular dissemination or systemic haematogenous spread by tumours which are typically advanced at presentation[15,16]. Equally, mucinous carcinoma may develop within an unrecognized rectal duplication and present as a perianal mass or discharge[17,18].

Viable tumour cells which represent clones capable of transplantation and proliferation have been retrieved from the lumen of the large intestine distal to established rectal tumours[18]. In this case it is believed that direct implantation of malignant cells occurred onto an area of anal granulation tissue within the fistula; a phenomenon which has previously been reported in patients with fresh haemorrhoidectomy wounds where a rectal carcinoma had been missed[19] and following local anal canal trauma following insertion of an endorectal stapler during low anterior resection[20]. Colorectal tumour implantation at the time of surgery is controversial and may possibly be diminished by the use of intraluminal cytocidal agents[21]. It would seem most likely in our case, given the long-standing nature of the perianal fistula, the similarity of histology of the two tumours, the intervening normal rectal mucosa and the stage of the colorectal primary. Evidence for this hypothesis has come from Scott *et al.*[7] where tumours shared DNA ploidy on flow cytometric analysis, suggesting a common clonal origin and from Hyman and Kida[10] where the pattern of cytokeratins 7 and 20 immunostaining of the anal tumour matched that of the colonic cancer.

## Conclusion

The management of this patient is unclear since too few cases have been reported to provide any evidence base for therapy. In our case, we performed a sphincter saving procedure as opposed to that of Hyman and Kida who recommended an extended abdominoperineal resection[22] The rôle of adjuvant chemoradiation or even sequential block dissection of the inguinal glands is controversial as is the place of supplemental brachytherapy. It is of some importance to define the precise aetiology of the tumour located within the perianal fistula, since primary mucinous adenocarcinoma of the anal ducts has a generally poor survival whereas those mucinous tumours which have presented as implantation metastases appear to have an overall favourable prognosis if treated by wide surgical excision. This case also highlights the ongoing need to biopsy all fistulae-in-ano, particularly where there has been an inherent change in the clinical features of the fistula such as an increase in discharge, bloody discharge, persistent pain or nodule formation.

NS: Assisted in the photography of the case and in the literature search

PP: Assessed the histology and the photomicrographs

AC: Assisted in the format and design of the paper

APZ: Assisted in the literature search and the writing of the article

All authors read and approved the final manuscript.
